# Diet replacement with whole insect larvae affects intestinal morphology and microbiota of broiler chickens

**DOI:** 10.1038/s41598-024-54184-9

**Published:** 2024-03-21

**Authors:** Stylianos Vasilopoulos, Ilias Giannenas, Ifigeneia Mellidou, Ioanna Stylianaki, Efthimia Antonopoulou, Athina Tzora, Ioannis Skoufos, Christos G. Athanassiou, Elias Papadopoulos, Paschalis Fortomaris

**Affiliations:** 1 Laboratory of Nutrition, Faculty of Veterinary Medicine, Aristotle University of Thessaloniki, 54124 Thessaloníki, Greece; 2 Institute of Plant Breeding and Genetic Resources, Hellenic Agricultural Organization-DIMITRA, 57001 Thessaloníki, Greece; 3 Laboratory of Pathology, Faculty of Veterinary Medicine, Aristotle University of Thessaloniki, 54124 Thessaloníki, Greece; 4 Laboratory of Animal Physiology, Department of Zoology, School of Biology, Aristotle University of Thessaloniki, 54124 Thessaloníki, Greece; 5 Laboratory of Animal Health, Food Hygiene and Quality, Department of Agriculture, University of Ioannina, 47100 Arta, Greece; 6 Laboratory of Animal Science, Nutrition and Biotechnology, School of Agriculture, University of Ioannina, 47100 Arta, Greece; 7 Laboratory of Entomology and Agricultural Zoology, Department of Agriculture, Crop Production and Rural Environment, University of Thessaly, Phytokou Str., 38446 N. Ionia, Volos, Greece; 8 Laboratory of Parasitology and Parasitic Diseases, Faculty of Veterinary Medicine, Aristotle University of Thessaloniki, 54124 Thessaloníki, Greece; 9 Laboratory of Animal Husbandry, Faculty of Veterinary Medicine, Aristotle University of Thessaloniki, 54124 Thessaloníki, Greece

**Keywords:** Microbiome, Gastrointestinal system

## Abstract

Insect-based diets are gaining interest as potential ingredients in improving poultry gut health. This study assessed the dietary treatment with whole dried *Tenebrio molitor* larvae (TM) on broiler chickens’ gut microbiota and morphology. 120 Ross-308 broilers received treated diets with 5% (TM5) and 10% (TM10) replacement ratio in a 35-day trial. Intestinal histomorphometry was assessed, as well as claudin-3 expression pattern and ileal and caecal digesta for microbial community diversity. Null hypothesis was tested with two-way ANOVA considering the intestinal segment and diet as main factors. The TM5 group presented higher villi in the duodenum and ileum compared to the other two (*P* < 0.001), while treated groups showed shallower crypts in the duodenum (*P* < 0.001) and deeper in the jejunum and ileum than the control (*P* < 0.001). Treatments increased the caecal Firmicutes/Bacteroidetes ratio and led to significant changes at the genus level. While *Lactobacilli* survived in the caecum, a significant reduction was evident in the ileum of both groups, mainly owed to *L*. *aviarius. Staphylococci* and *Methanobrevibacter* significantly increased in the ileum of the TM5 group*.* Results suggest that dietary supplementation with whole dried TM larvae has no adverse effect on the intestinal epithelium formation and positively affects bacterial population richness and diversity.

## Introduction

Insect meals are nowadays regarded as novel feeds, despite the fact that birds have always been pecking around for insects in their natural habitats. Given that insects have a natural role in the diets of numerous farmed livestock species, their role as feed has been reconsidered in recent years^1^.

In previous years, commercial broilers were selected based on a higher growth rate, as this was directly correlated with feed intake efficiency^2^. Nowadays, breeders still focus on direct selection for feed utilisation efficiency. Feed cost, on the other hand, constitutes about 60–70% of the total cost of poultry production^3^. Since significant poultry feed components, such as maize and soybean, are not only consumed by human beings and animals but also utilised in high quantities for other commercial purposes (e.g., oils, biofuel and other industrial products)^4^, feed efficiency improvement can contribute to production cost reduction^5^. Despite the significant improvement that has been achieved in FCR, however, 40–45% of waste is still generated^6^, necessitating the use of specific feed additives that have been found to improve feed efficiency. These substances continuously intensify their role in poultry diets, reducing production costs while allowing the utilisation of maize and soybean in other streams^6^. Furthermore, the impact of feed efficiency has not been equally studied compared with other specific indicators in poultry production, such as growth and body composition^5^. Novel feedstuffs or feed additives with bioactive compounds enhancing feed efficiency are required, as well as novel approaches, such as multi-omic analyses (whole-genome sequencing, meta-transcriptomics, metabolomics), in order to intensify the growth rate of chickens through the selection of genotypes showing improved feed efficiency^7,8^.

In this context, the inclusion of insect meal in poultry feeds has shown significant advantages as a protein alternative to current commodities, such as soybean meal and fishmeal^9–11^. Moreover, the utilisation of by-products of the primary agrifood sector and their transformation into nutrient sources can be the advantage of these novel feed ingredients. Insects can play this role by converting underused by-products into high-value nutrients, effectively reducing environmental degradation while supporting sustainable food systems^12^.

*Tenebrio molitor* (TM, Coleoptera: Tenebrionidae) larvae are one of the most promising novel protein sources, since they can be easily reared on by-products from primary agricultural sources as substrates^13^. Having already proven their nutritional value as pet foods as well as in aquaculture, for the past 2 years they have been entering the massive world market of poultry, pigs and cattle, due to their crude protein content and their amino acid and fatty acid profile^14^. Consequently, their high nutritional value, low rearing complexity and their importance in circular economy constitute TM larvae an important alternative protein source in a continuously growing feed industry.

Insects are efficient converters of energy and feed^15^ and their digestibility is considered high, especially when the chitin, ash and fibre content is low^11,15^. Their cost reduction potential expected to yield results in the forecoming years can be a turning point for the feed industry, proving as a dietary strategy that can lower both the feeding cost and the environmental impact of broiler farming^16^. Moreover, several changes in the intestinal bacterial composition may be due to the chitin content and the higher protein or fat content^17,18^. Thus, insect meals, in normal or defatted form, can be evaluated as animal feeds or, in smaller quantities, as feed additives. Their use as feedstuffs^19^ presents many advantages as they can be easily converted into powder form or pellets, forming a well digested feed for poultry.

Literature refers to TM larvae as an alternative protein source for broilers at various inclusion levels ranging from 1% up to a complete replacement of soymeal^20–22^, with varying effects. Biasato et al.^23^ have experimented with insect larvae meal at inclusion levels of 5, 10 and 15% in isonitrogenous and isoenergetic diets, showing a negative impact on Vh and Cd at high inclusion TM levels, compared with the Control and the TM 5% groups. Other researchers have studied the partial or full replacement of soybean with insect larvae meal, without any significant effects on growth performance, physicochemical properties and most carcass traits, but with an impact on the gastrointestinal tract (GIT), probably due to the chitin content of larvae meals^24^. Benzertiha et al. on the other hand have concluded that small additions of full-fat insect meals in the region of 0.2–0.3% in broiler chicken diets can help regulate the GIT’s microbiota and eventually improve their growth performance through the modulation of the immune system^22^, providing an effective role as an alternative growth promoting agent. Various studies on the caecal microbiota of broiler chickens fed on diets with increasing levels of TM larvae at 5, 10 and 15%, have provided a detailed characterization^23,25,26^.

The health of GIT impacts animal productivity. The gut’s role in body health and growth is widely accepted to be expressed through various functions, including protection against pathogens, absorption of nutrients or immune system maturation and the establishment of gut microbiota. Apparently, gut microbiota is associated with the environmental conditions and, in general, the health of birds^27^ and, therefore, affects gut morphology, digestion, nutrient absorption, vitamin synthesis, the production of short-chain fatty acids as well as the accumulation of pathogenic bacteria in the intestine^28^.

The present study was designed to evaluate the effects of a partial-replacement of diets with dried whole TM larvae on broiler chickens’ intestinal morphology and microbial ecosystem. The rationale of such an evaluation, is that the use of insects in bird diets can bring about effects beyond performance, that may be associated with GIT health indices.

## Methods

### Animal and sampling

The experimental protocol was approved by the Research and Ethics Committee of the School of Veterinary Medicine, Faculty of Health Sciences of the Aristotle University of Thessaloniki (GA, NR 776/17-12-2019). Husbandry, experimental and euthanasia procedures were conducted in appropriate research facilities; biosecurity precautions were taken according to the Greek legislative framework related to experimental animals and were approved by the local Public Veterinary Authorities (Reg. 489181(3254)/07.02.2018). Methods for the experimental design, sampling and results’ analysis and processing were in accordance with the ARRIVE guidelines and Good Farming Practice Guidelines were taken into consideration based on the Directive 2010/63/EC and the Commission recommendation 2007/526/EC. TM larvae were euthanized by freezing at very low temperatures (< − 60 °C) for 24 h in an Ultra-Low Temperature Freezer (DW-HL538, Zhongke Meiling Cryogenics Ltd). Prior to slaughter, broiler chickens were euthanised by electrical stunning procedures with the aid of a VE Memory stunner (FAF, Saint-Sernin-Sur-Rance, France).

A total of 120 1-day-old male Ross-308 chicks were randomly allotted to three dietary treatments, with four replicates of ten birds each. Each replicate was housed in a separate floor pen, equipped with an infrared lamp for heating, a feeder and nipple drinkers. The facility is located at the Research Institute of Animal Science, Hellenic Agricultural Organisation-DEMETER in Greece. The experimental protocol has been analytically described in a previous paper^29^. The basal diet was replaced with dried whole TM larvae at 5% (TM5 group) and 10% (TM10 group) to form the two experimental treatments (TM5 had 95% and TM10 had 90% of the basal diet remaining). Details of the diets can be found in Table 1. Marking the end of the experimentation on day 35, humane conditions were employed to euthanise the birds. Following euthanasia, two birds were randomly selected from each pen, followed by defeathering; intestinal samples were acquired for further microbiological and histological analysis.

**Table 1:** Broiler chicken basal and experimental diet composition and nutrient content, with 5% (TM5) and 10% (TM10) inclusion of dried whole *T. molitor* larvae (g/100gr feed).

Period			
Starter	Control	TM5	TM10
*Ingredients*, *%*		*Days 1–14*	
Maize	55.50	52.73	49.95
Soybean meal	35.77	33.97	32.19
Soybean oil	3.50	3.33	3.15
Palm fat	0.00	0.00	0.00
Calcium phosphate	1.46	1.38	1.31
Limestone (Calcium carbonate)	1.86	1.77	1.67
Salt	0.28	0.27	0.25
Sodium carbonate	0.21	0.20	0.19
Lysine	0.41	0.39	0.37
Methionine	0.39	0.37	0.35
Threonine	0.22	0.21	0.20
Valine	0.15	0.14	0.14
Vitamin and mineral premix*	0.25	0.24	0.23
*Tenebrio* *molitor* larvae	0.00	5.00	10.00
Total	100.00	100.00	100.00
Measured nutrient content, %			
Moisture	10.67	10.24	10.48
Protein As is	21.06	22.94	25.65
Fiber As is	2.99	3.63	4.12
Fat As is	4.66	6.01	7.72
Ash As is	6.38	6.18	6.27
Starch As is	39.93	36.99	33.79
Sugars, total As is	3.43	3.73	3.76
Calcium As is	2.07	2.27	2.46
Phosphorus As is	0.93	0.77	0.73

Grower	Control	TM5	TM10
Ingredients, %		Days 15–28	
Maize	60.00	57.00	54.00
Soybean meal	30.70	29.16	27.62
Soybean oil	3.50	3.33	3.15
Palm fat	1.00	0.95	0.90
Calcium phosphate	1.33	1.26	1.20
Limestone (Calcium carbonate)	1.68	1.60	1.50
Salt	0.23	0.22	0.21
Sodium carbonate	0.21	0.20	0.19
Lysine	0.40	0.38	0.36
Methionine	0.35	0.33	0.32
Threonine	0.21	0.20	0.19
Valine	0.14	0.13	0.13
V itamin and mineral premix*	0.25	0.24	0.23
*Tenebrio molitor* larvae	0.00	5.00	10.00
Total	100.00	100.00	100.00
Measured nutrient content, %			
Moisture	11.10	10.60	10.32
Protein As is	20.44	21.90	24.97
Fiber As is	2.57	3.16	3.96
Fat As is	5.87	7.19	8.82
Ash As is	5.91	5.66	5.71
Starch As is	40.06	37.29	33.34
Sugars, total As is	4.24	4.19	4.27
Calcium As is	1.32	1.83	2.50
Phosphorus As is	0.53	0.72	0.70

Finisher	Control	TM5	TM10
Ingredients, %		Days 29–35	
Maize	61.00	57.95	54.90
Soybean meal	28.62	27.18	25.75
Soybean oil	4.50	4.28	4.05
Palm fat	1.50	1.43	1.35
Calcium phosphate	1.28	1.22	1.15
Limestone (Calcium carbonate)	1.53	1.45	1.37
Salt	0.23	0.22	0.21
Sodium carbonate	0.19	0.18	0.17
Lysine	0.35	0.33	0.32
Methionine	0.31	0.29	0.28
Threonine	0.15	0.14	0.14
Valine	0.09	0.09	0.08
Vitamin and mineral premix *	0.25	0.24	0.23
*Tenebrio molitor* larvae	0.00	5.00	10.00
Total	100.00	100.00	100.00
Measured nutrient content, %			
Moisture	10.87	10.37	10.85
Protein As is	20.73	22.93	23.89
Fiber As is	2.60	3.08	3.81
Fat As is	6.40	7.76	9.35
Ash As is	5.74	5.64	5.55
Starch As is	40.52	35.17	28.91
Sugars, total As is	3.49	3.39	3.56
Calcium As is	1.84	1.85	2.04
Phosphorus As is	0.62	0.86	1.07

*Supplying per kilogram feed: 12,000 IU vitamin A, 5000 IU vitamin D_3_, 30 mg vitamin E, 3 mg vitamin K, 3 mg thiamine, 7 mg riboflavin, 6 mg pyridoxine, 0.035 mg vitamin B_12_, 40 mg niacin, 13 mg pantothenic acid, 1.5 mg folic acid, 0.13 mg biotin, 340 mg choline chloride, 55 mg Zn, 155 mg Mn, 20 mg Fe, 12 mg Cu, 0.2 mg Co, 1 mg I, 0.2 mg Se, and phytase 0.01 g.

### Histological staining and immunohistochemistry

Gava et al. methodology for the morphometric analysis of the small intestine was used^30^. On day 35 of the trial and shortly after euthanasia (2–5 min), tissue samples from the duodenum (middle part of the duodenal loop), jejunum (before Meckel’s diverticulum) and ileum (2 cm before the segment located between the caeca) were collected, fixed in a 10% neutral-buffered formalin solution, embedded in paraffin, cut into 3–4 μm cross-sections and stained with haematoxylin and eosin. Alcian Blue p.H 2.5/PAS staining (staining kit Bio-Optica, 04-163802A) was performed to detect goblet cells. Ten well-orientated and intact villus heights (Vh) (the distance from the tip of the villus to the villus–crypt junction) and crypt depths (Cd) (the depth of the invagination between adjacent villi) of each intestinal cross-section, the lamina propria width, the Vh/Cd ratio and the number of goblet cells per villus were recorded using the ImageJ image processing and analysis program (NIH, Bethesda, MD, J 1.53 k).

Tight junction (TJ) protein expression was assessed in mucosal epithelium using immunohistochemistry. After deparaffinization, endogenous peroxidases were blocked using a 0.3% H_2_O_2_ solution for 30 min. Antigen retrieval was achieved by incubation in Tris–EDTA buffer (pH 9.0) at 98 °C for 30 min. Primary monoclonal antibody against claudin-3 (rabbit polyclonal antibody specific to claudin-3, ab15102, Abcam, Cambridge, UK) was obtained and incubated in 4 °C overnight, and universal secondary antibody (BioGenex Super Sensitive™ (SS) Link-Label IHC Detection System) was used for staining. Its expression was measured with IHC separately in crypts and villi. A quantitative scoring system was used to evaluate claudin-3 expression levels (0–100%), according to Cuccato et al.^31^.

### Intestinal microbiota

#### DNA extraction, amplification and sequencing of the 16S rRNA gene

200 mg (on a wet-weight basis) of 36 homogenized caecal and ileal digesta samples taken from broiler chickens that were fed with the three diets (Control, 5% insects and 10% insects; with 6 biological replicates each one) were frozen at − 20 °C for DNA isolation. The total bacterial genomic DNA was extracted with the Qiagen DNeasy PowerSoil Pro Kit (QIAGEN, Carlsbad, USA), following manufacturer’s instructions. The quantity of the extracted DNA was measured using a Thermo Scientific™ NanoDrop™ (Thermo Fisher Scientific, Waltham, MA, USA), and its quality by agarose gel electrophoresis. DNA samples were stored at − 80 °C before further analysis.

The intestinal microbiota was assessed by sequencing the PCR amplified V3–V4 hypervariable region of the bacteria 16S rRNA using the primer pair 341F/806R (341F: 5′-CCTAYGGGRBGCASCAG-3′, 806R: 5′-GGACTACCVGGGTATCTAAT-3′), according to methodology analytically described in detail by Biasato et al.^25^ and Klindworth^32^. PCR amplification was performed using the primers mentioned above and the amplified 16S rDNA amplicons from each sample were paired-end sequenced (2 × 250) on the Illumina NovaSeq 6000 platform according to the 16S Metagenomic Sequencing Library Preparation protocol. The raw sequence data were successfully submitted to the NCBI SRA database (NCBI BioProject PRJNA899329).

### Statistical analysis, data processing and bioinformatics

Each pen (replicate) was considered the experimental unit. Differences in intestinal morphometry indices were tested at a 5% significance level through 2-way analysis of variance. The intestinal segment and the diet were used as fixed factors by applying the general linear model procedure (ANOVA/GLM). A full-factorial analysis with multiple comparison was carried out with a Bonferroni adjustment within each intestinal segment. Statistical analysis was conducted using the SPSS program (SPSS Statistics 27.0.1.0).

The raw paired-end reads were assigned to samples based on their unique barcodes and truncated by cutting off the barcode. In turn, paired-end reads were merged using FLASH (V1.2.7)^33^. Then, raw tags were filtered to maintain only the high-quality clean tags using the Quantitative Insights Into Microbial Ecology (QIIME, http://qiime.org/index.html) software package (V1.7.0)^34^. The tags were compared with the reference database (SILVA138 database) using the UCHIME algorithm (V 4.2.40)^35^ to detect and remove chimera sequences. The remaining high-quality and effective sequences were assigned to operational taxonomic units (OTUs) using the UPARSE (V7.0.1090, http://drive5.com/uparse/) pipeline^36^, with cluster defined at 97% sequence similarity.

For each representative sequence, species annotation was developed based on the Mothur method and the SILVA138 database (with a threshold of 0.8–1) for species annotation at each taxonomic rank^37,38^. OTUs abundance information were then rarefied to the lowest sequence count to provide an equal depth of sequence analysis for all diet groups. Subsequent alpha and beta diversity analyses were performed based on this normalized dataset.

Alpha-diversity (Chao1, Shannon, Observed-species) indexes were calculated with QIIME (Version 1.7.0) and displayed with the “phyloseq” package^39^ in R software (V2.15.3). Rarefaction metrics were computed using the alpha_rarefaction.py script in the QIIME package^40^. Heatmaps on the basis of the relative abundance of OTUs were generated using R^41^.

Beta-Diversity was also assessed with QIIME using principal coordinates analysis (PCoA) based on weighted and unweighted UniFrac distance matrix^42^. The Analysis of similarities (ANOSIM) was performed to assess the overall similarity among the different groups by testing the significance of spatial separation in PCoA. For the purpose of interpreting the distance matrix using average linkage, Unweighted Pair-group Method with Arithmetic Means (UPGMA) Clustering was performed, as a type of hierarchical clustering method and was conducted by QIIME software (Version 1.7.0). In order to illustrate the distribution of caecal and ileal bacterial communities among the three different diets, ternary plots were drawn using the R package (ggtern).

As a further step to unravel the microbial differences and identify the abundant taxa that were able to discriminate the different diets, the linear discriminant analysis (LDA) effect size (LEfSe) analysis was employed^43^. The Kruskal–Wallis sum test and the unpaired Wilcoxon test were applied, using an LDA score (log10) > 4, to detect the potential biomarkers.

## Results

### Histological staining and immunohistochemistry

The effects of part diet replacement with TM larvae on gut histomorphometric indices of broilers are shown in Table 2. Treatments incurred changes in the intestinal morphometry, with significant impact of both the intestinal segment (*P* < 0.001) and diet (*P* < 0.001) as well as their interaction (*P* = 0.008) on the Vh in the duodenum and ileum. Interestingly, these changes were more notable in the TM5 group compared to the ΤΜ10 group. Diet alone had a significant effect on the TM5 group (*P* < 0.001), while villi presented differences between the different intestinal segments, which was expected. On the other hand, the Cd was not affected by the fixed factors, that is the intestinal segment (*P* = 0.412) and diet (*P* = 0.222), it was, however, significantly affected by their interaction (*P* < 0.001). This was evident in the duodenum and jejunum, indicating that diet reduced the Cd in both treatments in the former, while incurring the opposite effect on the jejunum and mainly in the TM10 group.

**Table 2:** Effect of dietary replacement with whole *T. molitor* (TM) larvae on the intestinal epithelium morphology.

	Villus height		Crypt depth	
	Mean	SEM	Mean	SEM
Duodenum				
Control	704.58^b^	14.62	108.18^a^	5.41
TM5	790.07^a^		86.82^b^	
TM10	699.54^b^		83.34^b^	

Jejunum				
Control	611.71	14.62	82.45^b^	5.41
TM5	609.11		98.47^ab^	
TM10	607.35		113.52^a^	

Ileum				
Control	493.06^b^	14.62	89.70	5.41
TM5	567.18^a^		93.40	
TM10	494.47^b^		96.93	
Source	Probabilities			
Diet	< 0.001		0.222	
Intestinal Segment	< 0.001		0.412	
Interaction	0.008		< 0.001	

TM10, 10% TM larvae; TM5, 5% TM larvae.

*SEM* Standard error of mean.

Interaction, Effect of intestinal segment x Effect of diet.

^a–b^Means in the same row without superscripts in common differ significantly (Bonferroni; *P* < 0.05).

As a result, the Vh/Cd ratio dropped in the jejunum, whereas in the ileum, it increased in the TM5 group and decreased in the TM10 group. A partial influence of the diet on intestinal segments of the chickens was therefore evident, a finding that largely agrees with findings of previous studies^23,44^, especially with regard to Vh. No significant differences between treated groups and the control were detected.

Overall, sequencing of the V3–V4 hypervariable region of the bacterial 16S rRNA gene yielded a total of 3.57 M reads, with an average of 99,000 reads per sample and a median length of 419 bp (Suppl. Table 3). After data trimming and culling the low-quality reads, 3.05 M paired-end sequences, representing ∼85% of the total sequences, were acquired, with an average of 84,835 sequences per sample (Suppl. Table 4). After chimera removing, the high-quality sequences (in total 2,146,253) were classified into 2132 operational taxonomic units (OTUs; 97% identity), representing independent species belonging to 398 genera, 178 families, 104 orders, 43 classes, and 25 phyla (Suppl. Table 5).

### Alpha- and beta diversity

**Figure 1:**
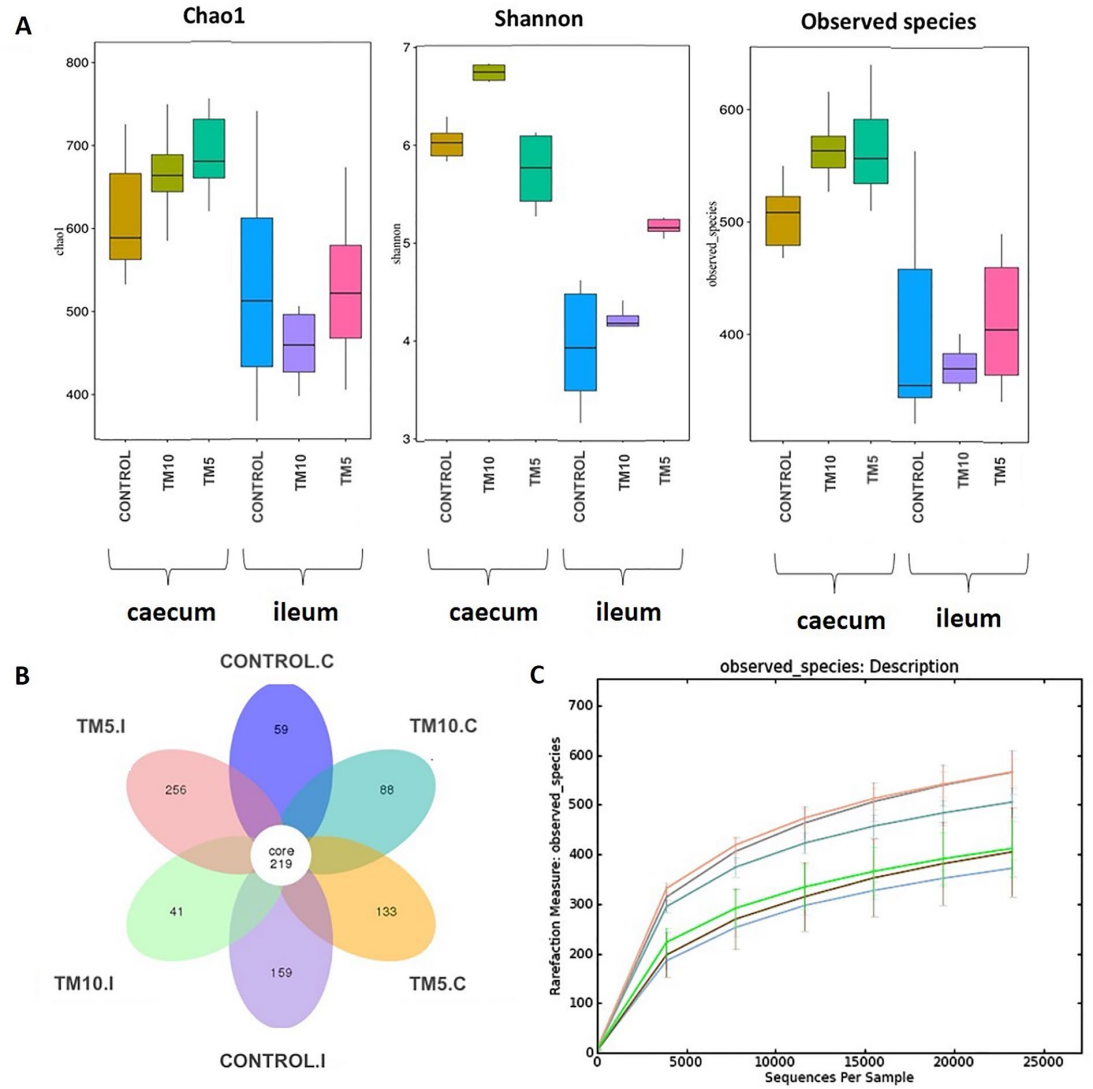
(**A**) Box plots of alpha-diversity (Chao1 value, Shannon index, observed species) of microbiome residing in the ileum and caecum of broilers in the three different diet groups. (**B**) Venn flower graph on the basis of the OTU number in different groups; (**C**) Rarefaction of the observed species. TM10, 10% insects; TM5, 5% insects; C, caecum; I, ileum.

As indicated by Chao 1, Shannon index and rarefaction of the observed species, the alpha-diversity highlighted greater species richness and diversity in caecal samples compared to ileal ones (Fig. 1A; Suppl. Table 6). Within caecal samples, chao1 diversity index revealed that community richness was substantially increased in the diets with insects, while between the two insect-enriched diets, there was a similar number of bacterial species. By contrast, ileal samples of the TM10 group showed the lowest species richness. Shannon index, which depicts species diversity within a group, was remarkably higher in the diets with 10% or 5% insects, in caecum and ileum, respectively, indicating fewer dominant species present in these groups compared to Control samples, which showed a higher abundance of predominant species. An interesting observation is that the TM5 group in both ileal and caecal samples had the greatest abundance of unique OTUs (Fig. 1B), which agrees with the number of observed species. This finding may be attributed to the presence of more dominant species with high abundance in the TM10 diets. The rarefaction curves on the basis of observed species indicated that the amount of sequencing data was sufficient for the analysis, illustrating a good coverage of the bacterial communities’ diversity (Fig. 1C).

**Figure 2:**
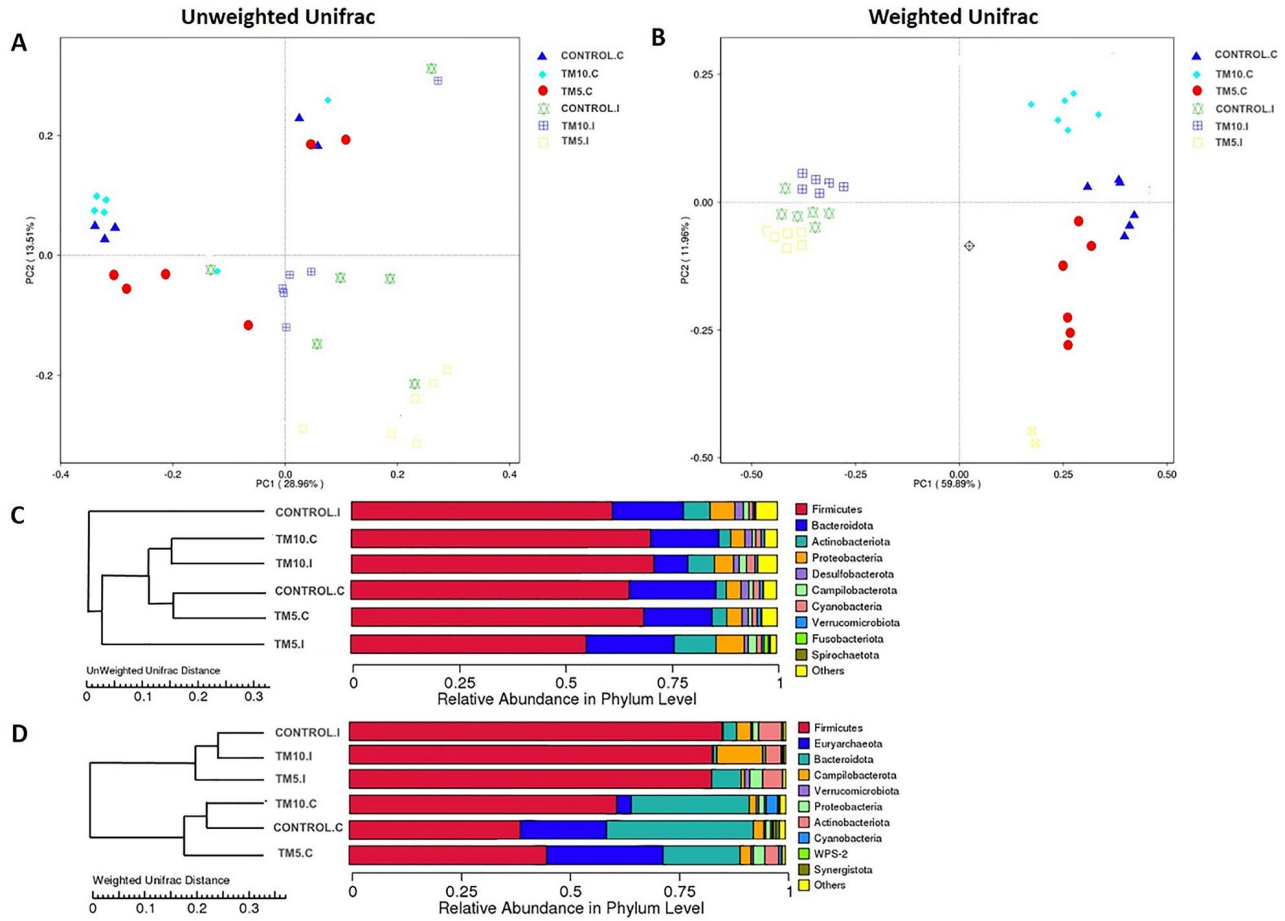
PCoA plots based on unweighted (**A**) and weighted (**B**) UniFrac distances of intestinal microbiome in the three different diet groups, generated using abundance at different taxonomic levels based on Bray–Curtis dissimilarities. UPGMA cluster tree based on Unweighted (**C**) and Weighted (**D**) Unifrac distance of the relative abundance of each sample by phylum. On the left, there is the UPGMA cluster tree structure, and on the right, there is the species relative abundance distribution at the phylum level for each sample. TM10, 10% insects; TM5, 5% insects; C, caecum; I, ileum.

Principal Coordinate Analysis (PCoA) plots of the overall diversity based on the unweighted and the weighted UniFrac metrics, using the UPGMA method (Unweighted Pair-group Method with Arithmetic Mean), are illustrated in Fig. 2 (A and B), in an attempt to explore differences/similarities between the different diets. A clear difference between ileal and caecal microbial communities was evident, explaining 42.5% and 71.9% of total variability based on unweighted and weighted Unifrac distances, respectively. It was also apparent that the clustering distances between samples were highly dependent on the tissue examined and to a lesser extent on the diet. Notwithstanding, on the basis of weighted Unifrac distances, results demonstrated that caecal samples of the group TM10 were clustered away from those of the TM5 and Control, suggesting distinct differences in microbial structure.

The clustering results were integrated with the species’ relative abundance column chart at phyla taxon level for each diet group (Fig. 2). The UPGMA trees based on Unweighted Unifrac distances showed that ileal Control samples were grouped separately from the other samples, reflecting a clear difference in microbial community (Fig. 2C). On the other hand, the phylogenetic tree based on weighted Unifrac distances revealed that the bacterial communities were divided into two main clusters in a tissue-specific manner, with each cluster containing samples from all the three diets (Fig. 2D). An interesting note, however, is that there was a relatively minor distance between Control and TM10 than between Control and TM5 groups, regardless of the tissue; this probably indicates a higher abundance of dominant species in these diets. It was also conceivable that the abundance of *Campilobacterota* as well as that of *Cyanobacteria* and *Actinobacteriota* in ileal and caecal samples, respectively, were able to distinguish the group of TM10 from the others.

### Sequencing data analysis and microbial diversity

**Figure 3:**
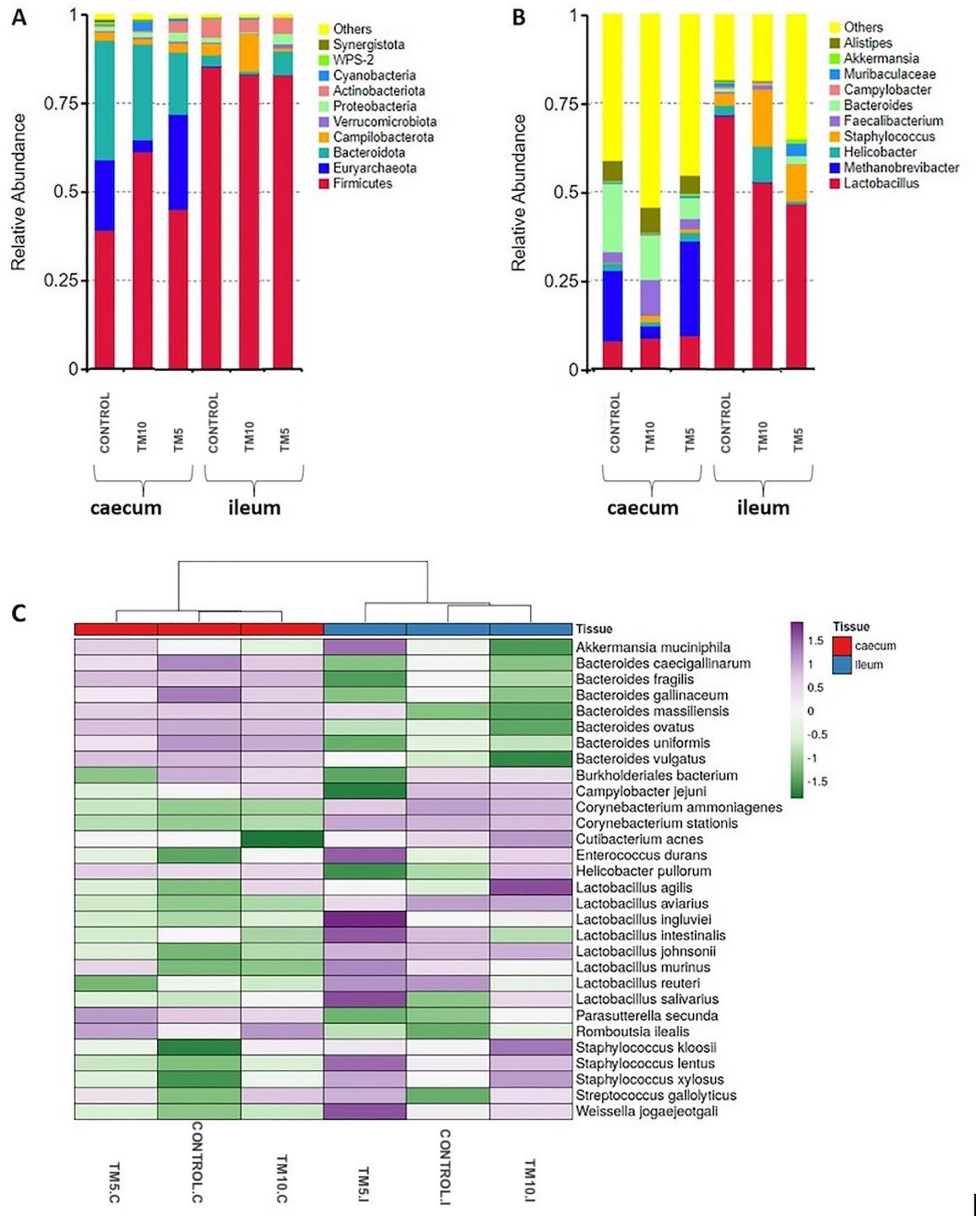
Distribution of the top 10 most abundant taxa of intestinal microbiota in ileum and caecum at the level of phylum (**A**), and genus (**B**). Heatmap with top 30 the taxonomic groups at the species level (**C**). TM10, 10% insects; TM5, 5% insects; C, caecum; I, ileum.

**Figure 4:**
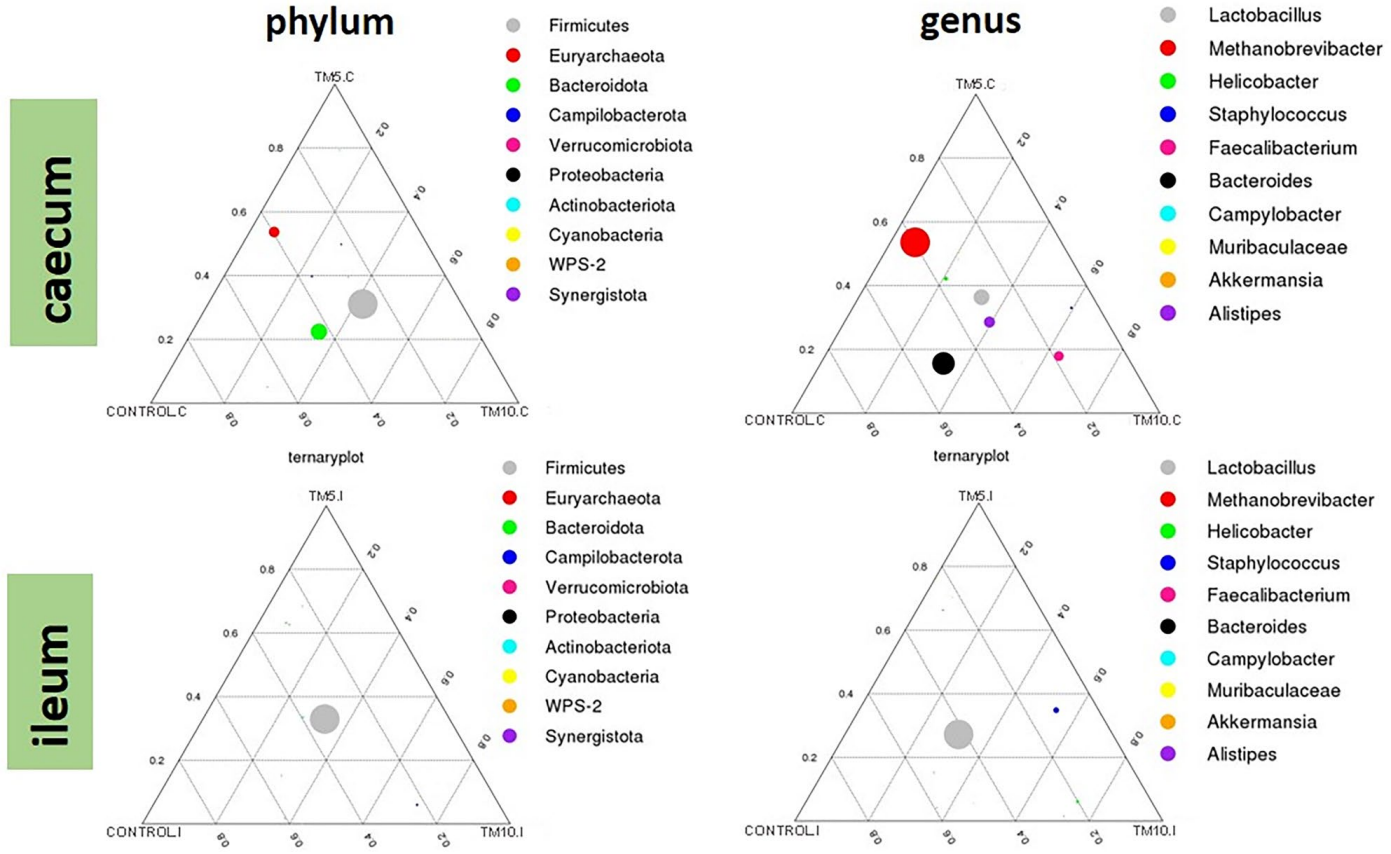
Ternary plots reveal OTUs relative abundance (dot size) at phylum and genus level among the three diet groups in caecum and ileum. Generalist taxa are represented as circles in the middle of the triangle, whereas sample-specific bacterial taxa are represented as circles in the summit or along the edges of the triangle.

**Figure 5:**
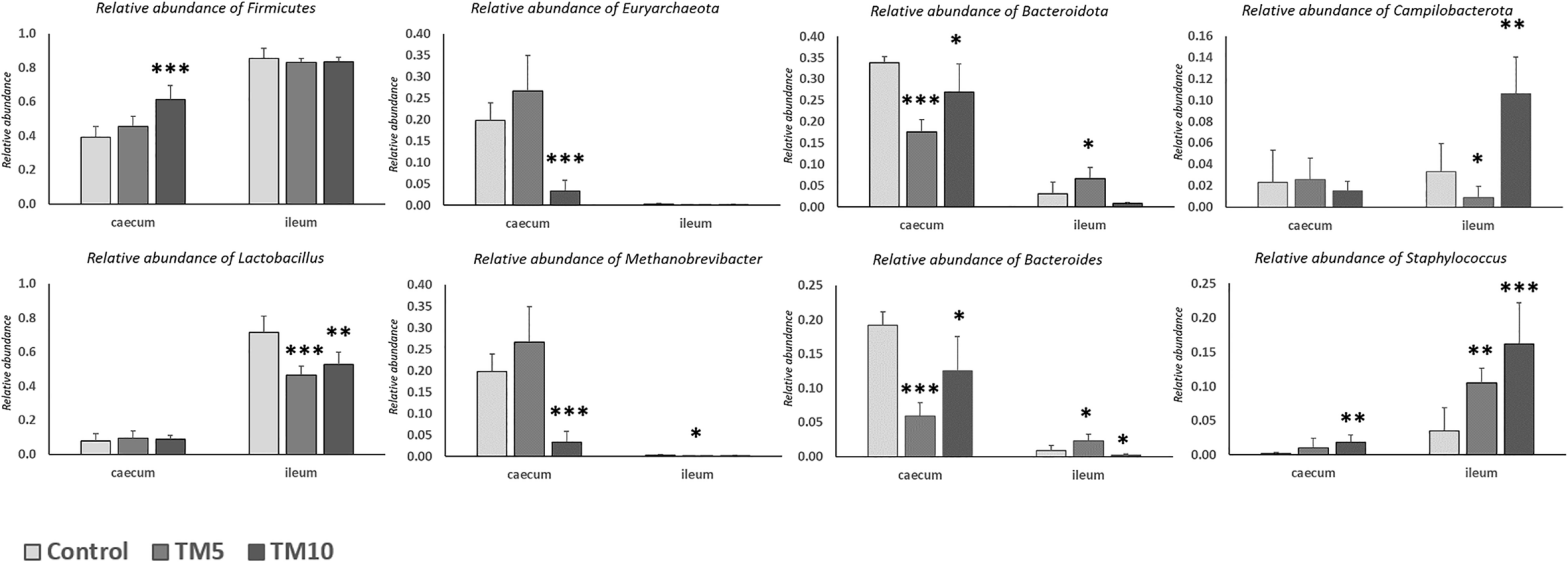
*P-values* for different microbiome phyla in the three trial groups at phylum (top) and genus (bottom) level. TM10, 10% insects; TM5, 5% insects. *: mean values differ significantly between them (*P* < 0.05); **: mean values differ significantly between them (*P* < 0.01); ***: mean values differ significantly between them (*P* < 0.0001).

At phylum level, intestinal microbiota communities were dominated by Firmicutes and Bacteroidetes, whereas Euryarcheota were mostly present and abundant in the caecum, regardless of the diet (Figs. 3A and 4). Other phyla that were also abundant included Actinobacteriota, Campilobacterota, and Proteobacteria. However, there were distinct differences between the caecum and the ileum, with Bacteroidetes dominating the former while Firmicutes comprised over 80% of the total microbial community of the latter (Suppl. Table 5). Concerning the caecum, a remarkable reduction in the relative abundance of Euryarchaeota and a relative increase in the quantity of Cyanobacteria was evident in the TM10 group. By contrast, the ileal samples of the TM10 group showed a noteworthy enhancement of Campilobacterota, and a decrease of Bacteroidetes compared with the TM5 and Control groups, as seen in Fig. 5.

At the genus level, a higher relative abundance of *Lactobacillus*, *Staphylococcus* and *Helicobacter* was evident in the ileal compared to the caecal samples, regardless the diet group (Figs. 3B and 4). On the contrary, the genera *Methanobrevibacter* (Archaea), *Faecalibacterium*, *Bacteroides* and *Alistipes* were more abundant in the caecum compared to the ileum. With regard to the differences in the microbial communities in the caecum that were related to the different diets, TM10 showed an increase in the abundance of *Faecalibacterium* and *Staphylococcus*, as well as a reduction in *Methanobrevibacter*, compared to the other groups. Accordingly, the diets of both TM5 and TM10 groups appeared to limit the abundance of *Lactobacillus* and stimulate *Staphylococcus*, while *Clostridium* was found increased in the TM10 group.

At the species level, plotting on a heatmap the top 30 taxa along the different diets, a higher relative abundance of several *Bacteroides* sp. as well as *Romboutsia ilealis* was observed in the caecum compared to the ileum, regardless the diet (Fig. 3C). Additionally, *Romboutsia ilealis, Enterococcus durans*, and several *Staphylococcus* sp. seemed to be associated with the caecal groups of broilers that were fed with insects. In the ileum, the TM5 group showed a high abundance of many *Lactobacilli* sp., including *Lactobacillus aviarius*, *Lactobacillus ingluviei*, and *Lactobacillus salivarius,* whereas the TM10 group increased the abundance of *Lactobacillus agilis*. Besides these differences in abundance at the species level, total *Lactobacilli* abundance was not altered along the different diets.

In line with these findings, ternary plots also depicted the generalists and group-specific bacterial taxa among the three diets. At the genus level, we observed that *Lactobacillus* and *Alistipes* were generally present in all caecal samples, thus considered as generalists (Fig. 4). One OTU identified as *Bacteroides* seemed to be specific for the Control diet, while the abundance of *Staphylococcus* seemed to discriminate the TM10 group (high abundance) from the Control diet (low abundance). Similarly, the archaea genus *Methanobrevibacter* seemed to separate the TM10 group (low abundance) from the other two diets (high abundance). In the ileum, *Lactobacillus* was generally abundant in all diets, while *Staphylococcus* seemed to be specific for diets with insects. Another OTUs, *Helicobacter,* was specific for the TM10 group, with more than fivefold higher abundance compared to the Control diet.

At species level, *Lactobacillus aviarius* and *Lactobacillus johnsonii* were generally abundant in caecal and ileal samples, irrespective of the diet. Unlike, *Romboutsia ilealis* and *Bacteroides uniformis* showed the lowest and the highest abundance, respectively, in the Control diets of the caecum compared to the diets with insects. Another species*, Staphylococcus xylosus*, seemed to be specialist taxa for the insect diets, and in fact in a dose-dependent manner, i.e., higher levels of insects increased the abundance of this microbial taxa, in both caecal and ileal samples.

### LEfSe analysis to reveal dominant microbial taxa in the different diets

**Figure 6:**
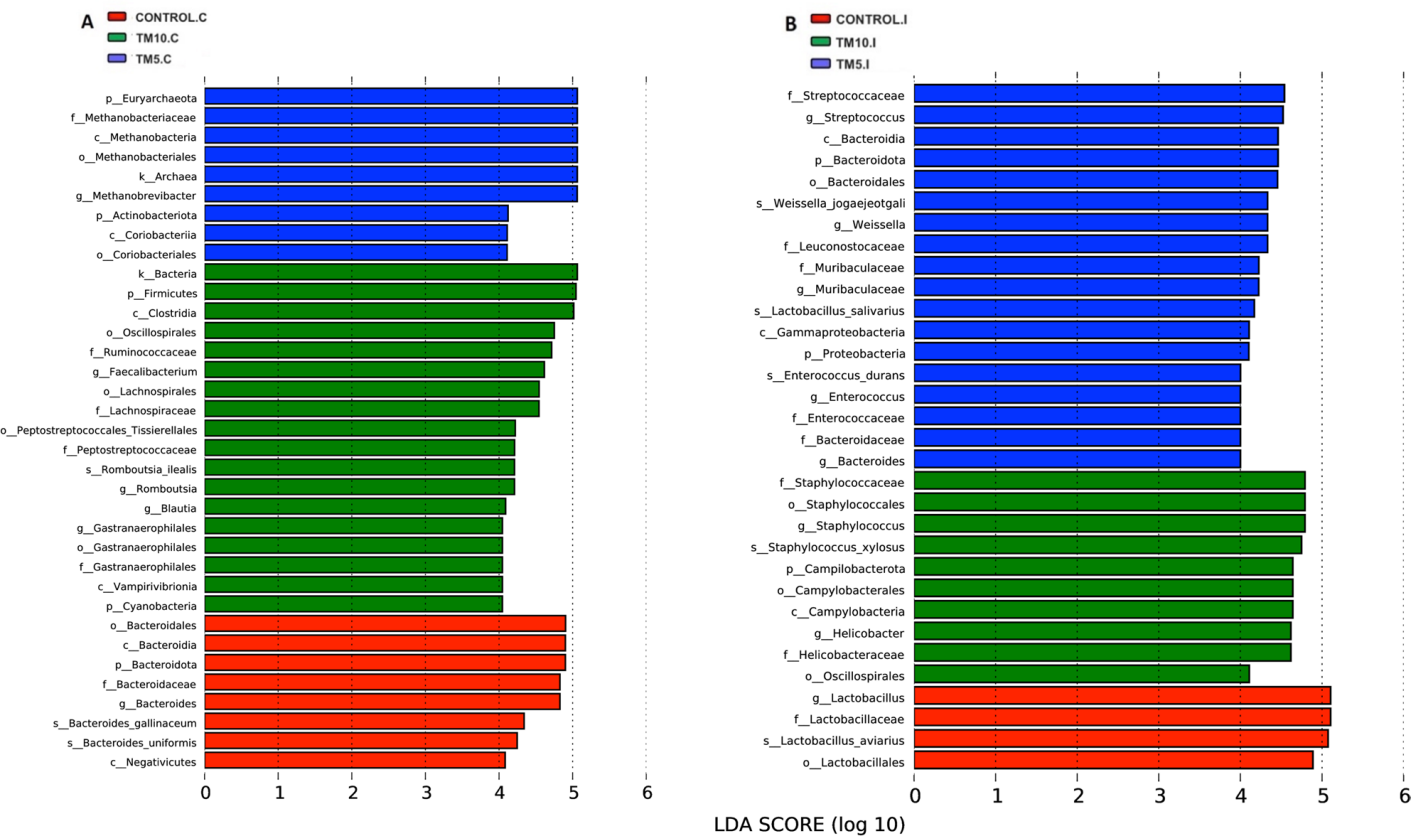
The differential phylogenetic distribution of bacteria in the caecum (**A**) and in the ileum (**B**) between the three different diets, on the basis of linear discriminant analysis (LDA) score > 4. Different coloured nodes represent the different diets, i.e., red for Control, green for TM10, and blue for TM5. TM10, 10% insects; TM5, 5% insects; C, caecum; I, ileum.

LefSe analysis was employed to identify microbial taxa that account for the observed microbial diversification, thus representing potential markers among the different diets, showing main differences between the Control group and the groups fed the treated diets with TM larvae on 5 or 10% levels. The results showed that the number of dominant bacteria (with LDA score > 4) in the three groups of diet were 19 and 16 in caecal and ileal samples, respectively (Fig. 6). Furthermore, it was evident that TM10 and TM5 groups in the caecum and the ileum, respectively, exhibited a greater number of dominant taxa than Control groups. Particularly, in the caecum, along the TM5 group, the classes of *Methanobacteria* and *Coriobacteriia* were the dominant taxa. In contrast, in the TM10 group, there were the families of *Gastranaerophilales*, *Lachnospiraceae*, *Ruminococcaceae*, and *Peptostreptococcaceae* (Fig. 6A). Similarly, with regard to the ileum, the families of *Bacteroidaceae*, *Muribaculaceae*, *Enterococcaceae*, *Leuconostocaceae* and *Streptococcaceae*, were associated with the TM5 group. At the same time, those of *Helicobacteraceae* and *Staphylococcaceae* with the TM10 group (Fig. 6B). The biomarker abundance comparison chart between the different diet group is also presented in Suppl. Fig. 1 (caecum) and Suppl. Fig. 2 (ileum).

## Discussion

There is still scarce information on how dietary insect meal inclusion affects intestinal microbiota. Furthermore, there have been minimal systematic studies on gut health evaluation based on diet composition, intestinal barrier and intestinal microbiota in chicken fields^45,46^. Indeed, various studies have taken place regarding poultry fed with different feeds, some including insect larvae meals as well as certain bioactive modulatory compounds of theirs (e.g. chitin, antimicrobial peptides or lauric acid), corn gluten, essential oils, minerals and acids, showing promising results on chicken growth performance, immune system and intestinal microbiota balance^9,23,47^. TM larvae have been evaluated for their nutritional quality, showcasing a higher protein, essential amino acid, vitamin and mineral content than plants and a positive effect on monogastric animals’ growth performance and digestibility^48–50^, not many, though, have investigated the impact of TM larvae inclusion on intestinal microbiota and gut morphology of broiler chickens. Most experiments have focused on the caecum due to the characteristics related to its position, digestion, biochemical processes and its role on gut health and nutrition^46^, but the results for the ileum are ever scarcer. The current study is, to our knowledge, the first one to investigate the inclusion of whole TM larvae in chicken diets and their impact on the intestinal morphology and microbiota both in the caecum and the ileum.

### Intestinal morphometry

The structure of the crypts and villi is a very important element of the intestinal epithelium, indicating gut cell proliferation and absorption and contributing to its homeostasis^51^. Poor intestinal development may be associated with various diseases and reduced nutrient absorption; ideally, gut morphology should be characterised by long villi for better digestive enzymes’ operation and nutrient transportation and shallow crypts that ensure a prolonged life of the former^45^. Absorption of nutrients is promoted by longer villi and shallower crypts with greater surface area, that enhance intestinal cell maturation and digestive enzyme activity^52^. Moreover, a good indicator of the maturity and functional capacity of the enterocytes is the Vh/Cd ratio^53^.

This study revealed an overall positive effect on the intestinal epithelium, especially in lower inclusion levels, partly in line with previous trials involving TM meal inclusion^14,54^. Significant effects were observed on the Vh, Cd and effectively on the Vh/Cd ratio. Lower dietary replacement with dried whole TM larvae favoured villi length, whereas higher replacement affected crypts. Furthermore, the intestinal segments mostly impacted by the diet regarding the villi were the duodenum and ileum, while crypts were affected mainly in the duodenum and the jejunum.

Previous studies indicated greater morphological developments in both the duodenum and jejunum compared to the ileum, whereas this study confirmed the effect on the duodenum, ileum and partly the jejunum. Even so, results suggest that treated groups maintained the physiological intestinal development with the bigger changes positively affecting the duodenum, which is the primary place of physical, chemical and hormonal activity^55^. One of the major components of the intestinal barrier is the formation of TJ between epithelial cells. Claudin-3, is one of the TJ proteins isoforms that is expressed in the chicken intestinal epithelium^56,57^. TJs regulate the paracellular pathway and form a selective barrier for the passage of ions and molecules^58^. It is demonstrated that in some cases TJ proteins can be regulated also by changing their location in the enterocytes^58^. Although we showed no significant differences in the expression pattern of claudin-3, insect supplement influence in TJs activity cannot be excluded.

Iji et al. have described the jejunum as an important site for nutrient digestion^59^. The lack of adverse effects on the jejunum in the current trial, mainly in low inclusion levels, indicates no deterioration of the digestive system’s health with the inclusion of whole TM large in broiler diets and agrees with findings of other research works^23^; crypts in the jejunum are usually associated with high energy sparing in broilers, although they may demonstrate a very active intestinal epithelium regeneration process^60^. It is evident that further investigation is needed in order to safely indicate that TM larvae can contribute to better metabolism and nutrient absorption and to evaluate optimum replacement ratios.

### Intestinal microbiota

The current trial pointed out various changes in the intestinal microbiota of the chicken fed supplemented feeds. a-Diversity showed more observed species in treated groups. Total richness represented by the Chao1 index was higher, especially in the TM10 group while the Shannon index indicated greater evenness, particularly for the TM5 group. A richer and denser population of bacteria may be a possible marker for improved intestinal health or can help evaluate specific intraspecies interactions.

Firmicutes and Bacteroidetes were the prevalent phyla in the caecum, partly confirming results of other researchers who have pointed out their positive effect on feed digestion and the general benefit on animal health^25,61^. Contrary to other trials, though, where Protobacteria were prevalent, these relatively increased at higher inclusion levels remaining, however, at low numbers. Dietary replacement induced an increase in Euryarchaeota that was inversely related to Firmicutes. Euryarchaeota include the family *Methanobacteriacae*, which may cause negative impact with a high methane production^62^; a significant drop, however, was monitored after treatment of the TM10 group. According to Adámková et al.^63^, the drop in Bacteroidetes population may incur a change in the digestion of complex polysaccharides. Otherwise, chitin content, high protein or high fat content may also alter the composition of intestinal microbiota.

Findings for the ileum were very different, as Firmicutes presented high relative abundance in all groups, contrary to Euryarchaeota that were almost absent, or to Bacteroidetes that showed significant increase only in the TM5 group. A large increase in the relative abundance of Campilobacterota evident in TM10, however, could be explained by the richer protein and fat content of the supplemented diets, as this phylum is associated with carbohydrate independent metabolism and relates to energy production metabolic processes from proteins, amino acids and fatty acids^64^.

At the genus level, a remarkable finding was the higher relative abundance of the genus *Lactobacilli* in the caecum after treatment, with *Lactobacillus aviarius* and *Lactobacillus salivarius* being the most prevalent species, in accordance with previous studies^28,65^ and *Lactobacillus murinus* and *Lactobacillus agilis* showing a higher abundance. Since *Lactobacilli* are widely regarded as probiotics and are participating in various physiological functions in poultry, affecting growth performance, vitamin production and bile acid metabolism, their proliferation in the ileum casts a positive effect on nutrient absorption and an indicator of beneficial established microbiota^27^. Additionally, they play an important role in the intestinal permeability and immunity while retaining the epithelial barrier function^66^.

Increased numbers of the genus *Clostridium* were also evident in the TM10 group, which naturally develop in aerobic environments of hatcheries as birds grow and represent one of the main bacterial genera in the poultry caecum^67^. Hence, the increase of the genera *Clostridium*, *Lactobacillus*, *Oscillospira* and *Faecalibacterium*, evident mainly in the caecum of the TM10 group, may also play a positive role in the intestinal villi and crypt formation as well as in pathogen control and, consequently, contribute to improved nutrient absorption, through increased butyrate production. In support of this, De Maesschalck et al. have shown that *Lactobacilli* produce lactate on prebiotic substrates and can develop synergistic actions with other bacteria (*Lachnospiraceae* family) that consume it to produce butyrate in broilers^68,69^. In this way, better feed conversion and weight gain may be achieved in broiler chickens^45^.

The TM5 group also showed increased numbers of *Helicobacter*. The caecal microbiota of broilers fed normal and treated diets was colonised principally by *Methanobrevibacter*, *Bacteroides*, *Alistipes* and significantly fewer *Lactobacilli* than the ileum; these are predominant genera in the chicken intestine during other experiments^70,71^. Interestingly, the higher abundance of *Helicobacter* in theTM5 caecum relates to a hydrogen-removing capability, paving the way for the proliferation of other bacteria and for the birds to achieve better energy efficiency from the food^72^. There are, however, other commensal *Helicobacter* species found in the poultry GIT, such as *Helicobacter pullorum*, which may cause gastroenteritis to humans^25^. Another possible attribute of the *Helicobacter* genus is its negative effect on mucin synthesis with higher inclusion levels of TM meal, through their enzymatic properties of breaking down mucins, rendering the epithelium susceptible to permeability^73^. Staphylococci increased both in the caecum and ileum; this increase, however, is exclusively owed to the species *Staphylococcus xylosus*, *Staphylococcus kloosii* and *Staphylococcus lentus* and principally to the former, which have been reported to be limiting fatty acid oxidation and, therefore, to avoiding rancidity in meat products^74^.

In conclusion, dietary whole TM larvae supplementation affected intestinal microbiota and morphology of broilers, showing increased diversity and possibly indicating improved intestinal health, especially in the TM10 group. The use of whole TM larvae positively affected the caecal Firmicutes/Bacteroidetes ratio, which is linked to energy efficiency harvesting. At the genus level, a higher relative abundance of *Lactobacilli* was evident in the caecum, together with an increase in *Staphylococci* (*P* < 0.05), the latter, however, owed to species limiting fatty acid oxidation. Increased populations of specific genera, especially in the TM10 group, could be an indication of improved formation of the intestinal epithelium. Broilers maintained the physiological intestinal development, with a higher Vh/Cd ratio in the duodenum where primary physical, chemical and hormonal activities take place, without any adverse effects on the jejunum, especially at a low replacement level. In total, the 5% replacement had a greater effect on gut morphology compared to the 10%, mainly in the duodenum and ileum, suggesting that a lower replacement level may be preferable for broilers as a more influential feed additive. The results of this experiment indicate that TM larvae can be regarded as a promising feed ingredient for use in broiler nutrition, however, future experimentation would help reassess the colonisation of pathogenic bacteria, such as S*almonella* and *Campylobacter,* as they pose major health risks to humans and focus on preventative mechanisms by probiotic microorganisms against pathogenesis.

### Supplementary Information

Supplementary Figure 1.

Supplementary Figure 2.

Supplementary Table 1.

Supplementary Table 2.

Supplementary Table 3.

Supplementary Table 4.

Supplementary Table 5.

Supplementary Table 6.

## Data Availability

Data described in the manuscript, code book, and analytic code will be made publicly and freely available without restriction. The raw sequence data analysed during the current study on microbiota are available in the NCBI SRA database repository, NCBI BioProject PRJNA899329. Raw datasets of intestinal morphology used and/or analysed during the current study are available from the corresponding author on reasonable request.

## Abbreviations

Cd: Crypt depth
FCR: feed conversion ratio
GIT: Gastrointestinal tract
S.D.: Standard deviation
SEM: Standard error of the means
TJ: Tight junction
TM: *Tenebrio molitor*
TM5: Experimental group with 5% replacement of basal diet with whole *T. molitor* larvae
TM10: Experimental group with 10% replacement of basal diet with whole *T. molitor* larvae
Vh: Villus height

## References

[1] Sogari G, Amato M, Biasato I, Chiesa S, Gasco L (2019). The potential role of insects as feed: A multi-perspective review. Animals.

[2] Crawford, R. D. Poultry breeding and genetics. (1990).

[3] Singh M (2015). Performance and carcass characteristics of guinea fowl fed on dietary Neem (*Azadirachta*
*indica*) leaf powder as a growth promoter. Iran. J. Vet. Res.

[4] van Huis A (2022). Edible insects: Challenges and prospects. Entomol. Res..

[5] Prakash A (2021). Differential gene expression in liver of colored broiler chicken divergently selected for residual feed intake. Trop. Anim. Health Prod..

[6] Prakash A, Saxena VK, Singh MK (2020). Genetic analysis of residual feed intake, feed conversion ratio and related growth parameters in broiler chicken: a review. Worlds Poult. Sci. J..

[7] Chen F (2019). Transcriptome analysis of differentially expressed genes related to the growth and development of the Jinghai yellow chicken. Genes.

[8] Tous N (2022). Novel strategies to improve chicken performance and welfare by unveiling host-microbiota interactions through hologenomics. Front. Physiol..

[9] Gasco L, Finke M, Huis AV (2018). Can diets containing insects promote animal health?. J. Insects Food Feed..

[10] Van Huis A (2013). Potential of insects as food and feed in assuring food security. Annu. Rev. Entomol..

[11] Makkar HP, Tran G, Heuzé V, Ankers P (2014). State-of-the-art on use of insects as animal feed. Anim. Feed Sci. Technol..

[12] Murta, D. The future of animal feeding. *Insects as Animal Feed: Novel Ingredients for Use in Pet, Aquaculture and Livestock Diets*, 126–138 (2021). 10.5713/ab.22.0001B PMC8738934 34986298

[13] Rumbos CI, Karapanagiotidis IT, Mente E, Psofakis P, Athanassiou CG (2020). Evaluation of various commodities for the development of the yellow mealworm. Tenebrio molitor. Sci. Rep..

[14] Biasato I (2016). Effects of dietary *Tenebrio*
*molitor* meal inclusion in free-range chickens. J. Anim. Physiol. Anim. Nutr. (Berl).

[15] Sánchez-Muros M-J, Barroso FG, Manzano-Agugliaro F (2014). Insect meal as renewable source of food for animal feeding: A review. J. Clean. Prod..

[16] van Huis A (2022). Progress and challenges of insects as food and feed. New Aspects Meat Qual..

[17] Benzertiha A (2019). *Tenebrio*
*molitor* and *Zophobas*
*morio* full-fat meals in broiler chicken diets: Effects on nutrients digestibility, digestive enzyme activities, and cecal microbiome. Animals.

[18] Borrelli L (2017). Insect-based diet, a promising nutritional source, modulates gut microbiota composition and SCFAs production in laying hens. Sci. Rep..

[19] van Huis A, Gasco L (2023). Insects as feed for livestock production. Science.

[20] Ramos-Elorduy J, González EA, Hernández AR, Pino JM (2002). Use of *Tenebrio*
*molitor* (Coleoptera: Tenebrionidae) to recycle organic wastes and as feed for broiler chickens. J. Econ. Entomol..

[21] Bovera F (2015). Yellow mealworm larvae (*Tenebrio*
*molitor*, L.) as a possible alternative to soybean meal in broiler diets. Br. Poult. Sci..

[22] Benzertiha A (2020). *Tenebrio*
*molitor* and *Zophobas*
*morio* full-fat meals as functional feed additives affect broiler chickens' growth performance and immune system traits. Poult. Sci..

[23] Biasato I (2018). Yellow mealworm larvae (*Tenebrio*
*molitor*) inclusion in diets for male broiler chickens: Effects on growth performance, gut morphology, and histological findings. Poult. Sci..

[24] Bovera F (2016). Use of *Tenebrio*
*molitor* larvae meal as protein source in broiler diet: Effect on growth performance, nutrient digestibility, and carcass and meat traits. J. Anim. Sci..

[25] Biasato I (2019). Gut microbiota and mucin composition in female broiler chickens fed diets including yellow mealworm (*Tenebrio*
*molitor*, L.). Animals.

[26] Sedgh-Gooya S (2021). Yellow mealworm, *Tenebrio*
*molitor* (Col: Tenebrionidae), larvae powder as dietary protein sources for broiler chickens: Effects on growth performance, carcass traits, selected intestinal microbiota and blood parameters. J. Anim. Physiol. Anim. Nutr. (Berl).

[27] Aruwa CE, Pillay C, Nyaga MM, Sabiu S (2021). Poultry gut health–microbiome functions, environmental impacts, microbiome engineering and advancements in characterization technologies. J. Anim. Sci. Biotechnol..

[28] Xiao Y (2017). Microbial community mapping in intestinal tract of broiler chicken. Poult. Sci..

[29] Vasilopoulos S (2022). Growth performance, welfare traits and meat characteristics of broilers fed diets partly replaced with whole *Tenebrio*
*molitor* larvae. Anim. Nutr..

[30] Gava MS (2015). Determining the best sectioning method and intestinal segment for morphometric analysis in broilers. Braz. J. Poult. Sci..

[31] Cuccato M (2022). Assessment of antimicrobial effects on broiler gut barrier through histopathology and immunohistochemistry of tight-junction proteins. Front. Vet. Sci..

[32] Klindworth A (2013). Evaluation of general 16S ribosomal RNA gene PCR primers for classical and next-generation sequencing-based diversity studies. Nucleic Acids Res..

[33] Magoč T, Salzberg SL (2011). FLASH: Fast length adjustment of short reads to improve genome assemblies. Bioinformatics.

[34] Caporaso JG (2010). QIIME allows analysis of high-throughput community sequencing data. Nat. Methods.

[35] Edgar RC, Haas BJ, Clemente JC, Quince C, Knight R (2011). UCHIME improves sensitivity and speed of chimera detection. Bioinform..

[36] Edgar RC (2013). UPARSE: Highly accurate OTU sequences from microbial amplicon reads. Nat. Methods.

[37] Quast C (2012). The SILVA ribosomal RNA gene database project: Improved data processing and web-based tools. Nucleic Acids Res..

[38] Wang Q, Garrity GM, Tiedje JM, Cole JR (2007). Naive Bayesian classifier for rapid assignment of rRNA sequences into the new bacterial taxonomy. Appl. Environ. Microbiol..

[39] McMurdie PJ, Holmes S (2013). phyloseq: An R package for reproducible interactive analysis and graphics of microbiome census data. PLoS One.

[40] Kuczynski, J. *et al.* (Wiley, 2005).

[41] Ling Z (2014). Altered fecal microbiota composition associated with food allergy in infants. Appl. Environ. Microbiol..

[42] Lozupone C, Knight R (2005). UniFrac: A new phylogenetic method for comparing microbial communities. Appl. Environ. Microbiol..

[43] Segata N (2011). Metagenomic biomarker discovery and explanation. Genome Biol..

[44] Biasato I (2017). Effects of yellow mealworm larvae (*Tenebrio*
*molitor*) inclusion in diets for female broiler chickens: implications for animal health and gut histology. Anim. Feed Sci. Technol..

[45] Biasato I (2018). Modulation of intestinal microbiota, morphology and mucin composition by dietary insect meal inclusion in free-range chickens. BMC Vet. Res..

[46] Oakley BB (2014). The chicken gastrointestinal microbiome. FEMS Microbiol. Lett..

[47] Giannenas I (2017). Effects of protease addition and replacement of soybean meal by corn gluten meal on the growth of broilers and on the environmental performances of a broiler production system in Greece. PLoS One.

[48] De Marco M (2015). Nutritional value of two insect larval meals (*Tenebrio*
*molitor* and *Hermetia*
*illucens*) for broiler chickens: Apparent nutrient digestibility, apparent ileal amino acid digestibility and apparent metabolizable energy. Anim. Feed Sci. Technol..

[49] Józefiak A (2018). Full-fat insect meals as feed additive: The effect on broiler chicken growth performance and gastrointestinal tract microbiota. J. Anim. Feed Sci..

[50] Hong J, Han T, Kim YY (2020). Mealworm (*Tenebrio*
*molitor* Larvae) as an alternative protein source for monogastric animal: A review. Animals.

[51] Kwon O, Han T-S, Son M-Y (2020). Intestinal morphogenesis in development, regeneration, and disease: The potential utility of intestinal organoids for studying compartmentalization of the crypt-villus structure. Front. Cell Dev. Biol..

[52] Adeleye O (2018). Serum chemistry and gut morphology of two strains of broiler chickens to varying interval of post hatch feeding. Vet. Anim. Sci..

[53] Hampson D (1986). Alterations in piglet small intestinal structure at weaning. Res. Vet. Sci..

[54] Sedgh-Gooya S, Torki M, Darbemamieh M, Khamisabadi H, Abdolmohamadi A (2022). Growth performance and intestinal morphometric features of broiler chickens fed on dietary inclusion of yellow mealworm (*Tenebrio*
*molitor*) larvae powder. Vet. Med. Sci..

[55] Macari M (1998). Aspectos fisiológicos do sistema digestivo das aves. Vet. Sacavet-Semana Acad..

[56] Awad WA, Hess C, Hess M (2017). Enteric pathogens and their toxin-induced disruption of the intestinal barrier through alteration of tight junctions in chickens. Toxins.

[57] Ozden O (2010). Developmental profile of claudin-3,-5, and-16 proteins in the epithelium of chick intestine. Anatom. Record: Adv. Integr. Anat. Evolut. Biol..

[58] Roxas JL (2010). Enterohemorrhagic *E.*
*coli* alters murine intestinal epithelial tight junction protein expression and barrier function in a Shiga toxin independent manner. Lab. Investig..

[59] Iji P, Saki A, Tivey D (2001). Body and intestinal growth of broiler chicks on a commercial starter diet. 1. Intestinal weight and mucosal development. Br. Poult. Sci..

[60] Gottardo E (2016). Regeneration of the intestinal mucosa in Eimeria and *E.*
*Coli* challenged broilers supplemented with amino acids. Poult. Sci..

[61] Ndotono EW, Khamis FM, Bargul JL, Tanga CM (2022). Insights into the gut microbial communities of broiler chicken fed black soldier fly larvae-desmodium-based meal as a dietary protein source. Microorganisms.

[62] Lv J (2022). Effects of different probiotic fermented feeds on production performance and intestinal health of laying hens. Poult. Sci..

[63] Adámková A (2017). Welfare of the mealworm (*Tenebrio*
*molitor*) breeding with regard to nutrition value and food safety. Potravinarstvo slovak J. Food Sci..

[64] Polansky O (2016). Important metabolic pathways and biological processes expressed by chicken cecal microbiota. Appl. Environ. Microbiol..

[65] Dokou S (2023). A phytobiotic extract, in an aqueous or in a cyclodextrin encapsulated form, added in diet affects meat oxidation, cellular responses and intestinal morphometry and microbiota of broilers. Front. Anim. Sci..

[66] Xu C (2022). *Lactobacillus*
*salivarius* CML352 isolated from Chinese local breed chicken modulates the gut microbiota and improves intestinal health and egg quality in late-phase laying hens. Microorganisms.

[67] Rychlik I (2020). Composition and function of chicken gut microbiota. Animals.

[68] De Maesschalck C (2015). The effects of xylo-oligosaccharides on performance and microbiota in broiler chickens. Appl. Environ. Microbiol..

[69] Ducatelle R, Goossens E, Eeckhaut V, Van Immerseel F (2023). Poultry gut health and beyond. Anim. Nutr..

[70] Wei S, Morrison M, Yu Z (2013). Bacterial census of poultry intestinal microbiome. Poult. Sci..

[71] Costa MC (2017). Different antibiotic growth promoters induce specific changes in the cecal microbiota membership of broiler chicken. PLoS One.

[72] Sergeant MJ (2014). Extensive microbial and functional diversity within the chicken cecal microbiome. PLoS One.

[73] Duangnumsawang Y, Zentek J, Goodarzi Boroojeni F (2021). Development and functional properties of intestinal mucus layer in poultry. Front. Immunol..

[74] Leroy S, Vermassen A, Ras G, Talon R (2017). Insight into the genome of Staphylococcus xylosus, a ubiquitous species well adapted to meat products. Microorganisms.

