# Supplementary material for: Diet replacement with whole insect larvae affects intestinal morphology and microbiota of broiler chickens
Source: Sci Rep. 2024 Mar 21;14:6836. doi: 10.1038/s41598-024-54184-9 (PMC10957974; doi:10.1038/s41598-024-54184-9)
Supplement: Supplementary file 1 — Supplementary Figure 1. [file 41598_2024_54184_MOESM1_ESM.docx]

**Suppl. Fig. 1.** The distribution of caecal microbial taxa representing putative biomarkers along the three group of diets, according to LefSe analysis with linear discriminant analysis (LDA) score >4. TM10, 10% insects; TM5, 5% insects; C, caecum.

**
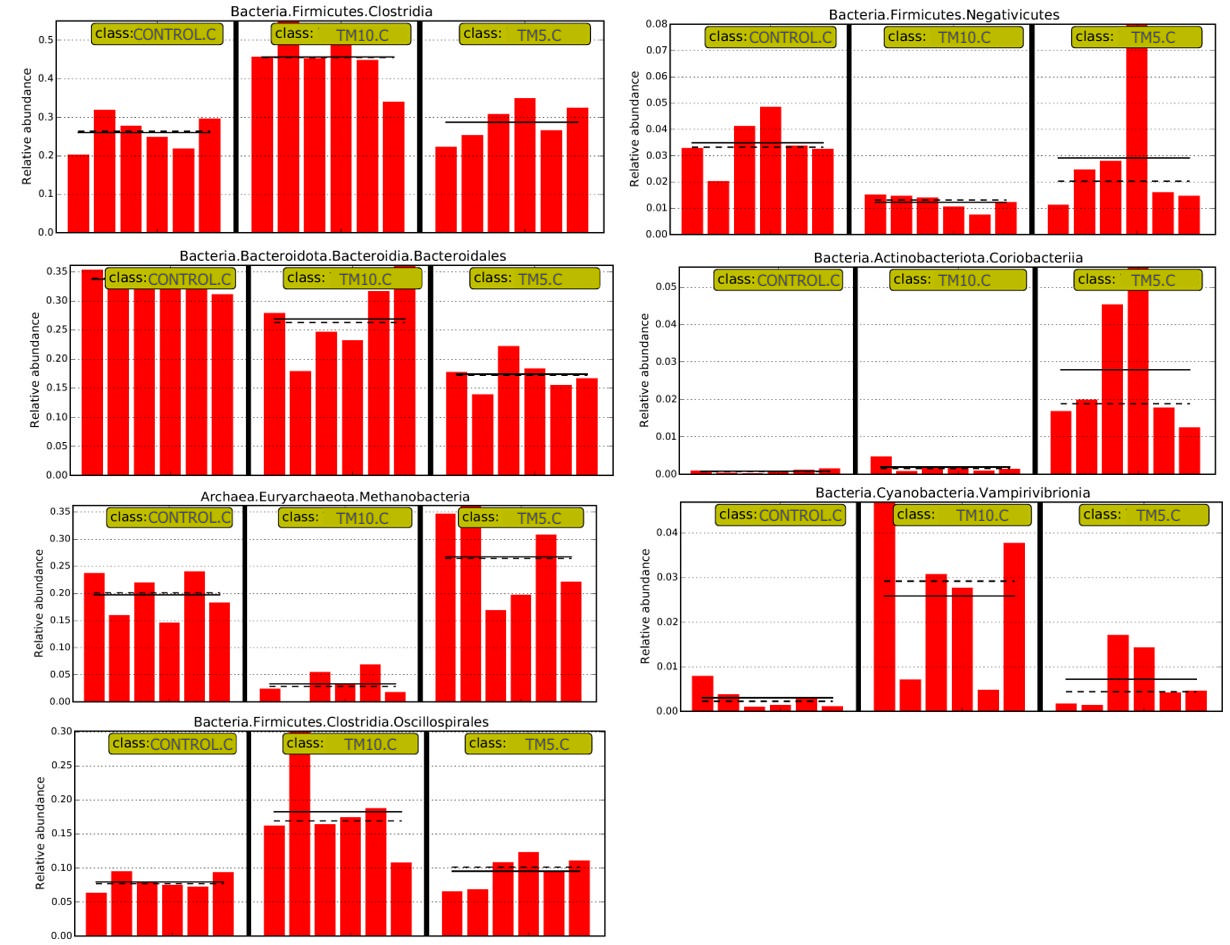
**
