# Supplementary material for: Diet replacement with whole insect larvae affects intestinal morphology and microbiota of broiler chickens
Source: Sci Rep. 2024 Mar 21;14:6836. doi: 10.1038/s41598-024-54184-9 (PMC10957974; doi:10.1038/s41598-024-54184-9)
Supplement: Supplementary file 2 — Supplementary Figure 2. [file 41598_2024_54184_MOESM2_ESM.docx]

**Suppl. Fig. 2.** The distribution of ileal microbial taxa representing putative biomarkers along the three group of diets, according to LefSe analysis with linear discriminant analysis (LDA) score >4. TM10, 10% insects; TM5, 5% insects; I, ileum.

**
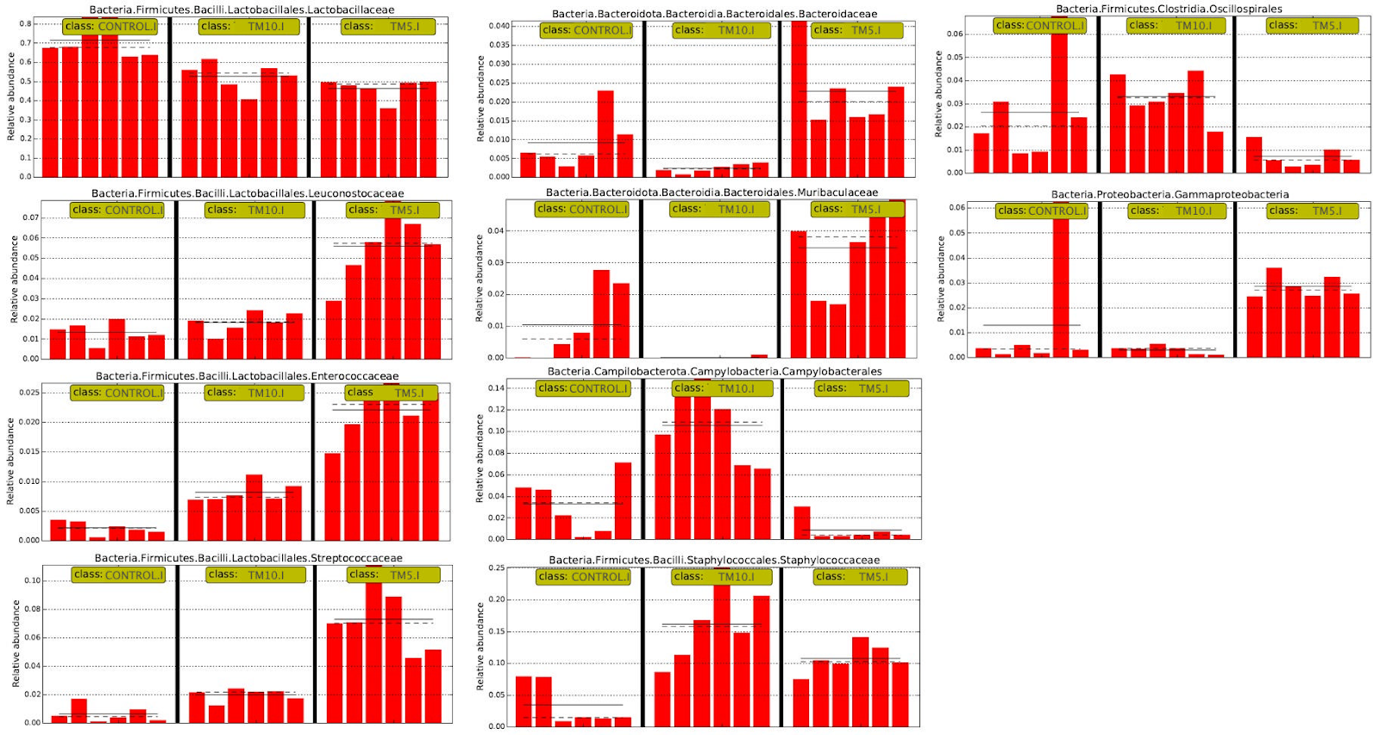
**
