# Supplementary material for: Diet replacement with whole insect larvae affects intestinal morphology and microbiota of broiler chickens
Source: Sci Rep. 2024 Mar 21;14:6836. doi: 10.1038/s41598-024-54184-9 (PMC10957974; doi:10.1038/s41598-024-54184-9)
Supplement: Supplementary file 3 — Supplementary Table 1. [file 41598_2024_54184_MOESM3_ESM.docx]

**Suppl. Table 1.** Broiler chicken basal diet (g/100gr feed).

| **Item** | **Basal diet** | | |
| --- | --- | --- | --- |
|  | **Starter period** | **Grower period** | **Finisher period** |
|  | **d 1–14** | **d 15–28** | **d 29–35** |
| Ingredients |  |  |  |
| Maize | 55.50 | 60.00 | 61.00 |
| Soybean meal | 35.77 | 30.70 | 28.62 |
| Soybean oil | 3.50 | 3.50 | 4.50 |
| Palm fat | 0.00 | 1.00 | 1.50 |
| Calcium phosphate | 1.46 | 1.33 | 1.28 |
| Limestone (Calcium carbonate) | 1.86 | 1.68 | 1.53 |
| Salt | 0.28 | 0.23 | 0.23 |
| Sodium carbonate | 0.21 | 0.21 | 0.19 |
| Lysine | 0.41 | 0.40 | 0.35 |
| Methionine | 0.39 | 0.35 | 0.31 |
| Threonine | 0.22 | 0.21 | 0.15 |
| Valine | 0.15 | 0.14 | 0.09 |
| Vitamin and mineral premix * | 0.25 | 0.25 | 0.25 |
| Total | 100.00 | 100.00 | 100.00 |
| Proximate analysis (As fed basis) |  |  |  |
| Moisture | 10.15 | 10.55 | 11.14 |
| Protein | 22.00 | 21.00 | 20.00 |
| Crude fibre | 2.85 | 2.65 | 2.55 |
| Crude fat | 4.84 | 6.11 | 6.65 |
| Ash | 6.12 | 5.65 | 5.58 |
| Calculated analysis (As fed basis) |  |  |  |
| Total energy, kcal/kg | 4,020.2 | 4,088.2 | 4,080.3 |
| Lysine | 1.41 | 1.28 | 1.15 |
| Methionine + Cystine | 1.08 | 0.99 | 0.92 |
| Methionine | 0.73 | 0.67 | 0.62 |
| Threonine | 0.98 | 0.89 | 0.79 |
| Tryptophan | 0.28 | 0.25 | 0.24 |
| Valine | 1.10 | 1.02 | 0.92 |

* Supplying per kilogram feed: 12,000 IU vitamin A, 5,000 IU vitamin D_3_, 30 mg vitamin E, 3 mg vitamin K, 3 mg thiamine, 7 mg riboflavin, 6 mg pyridoxine, 0.035 mg vitamin B_12_, 40 mg niacin, 13 mg pantothenic acid, 1.5 mg folic acid, 0.13 mg biotin, 340 mg choline chloride, 55 mg Zn, 155 mg Mn, 20 mg Fe, 12 mg Cu, 0.2 mg Co, 1 mg I, 0.2 mg Se, and phytase 0.01 g.
