# Supplementary material for: Diet replacement with whole insect larvae affects intestinal morphology and microbiota of broiler chickens
Source: Sci Rep. 2024 Mar 21;14:6836. doi: 10.1038/s41598-024-54184-9 (PMC10957974; doi:10.1038/s41598-024-54184-9)
Supplement: Supplementary file 4 — Supplementary Table 2. [file 41598_2024_54184_MOESM4_ESM.docx]

**Suppl. Table 2.** Effect of dietary supplementation with whole *T. molitor* (TM) larvae on broiler intestinal morphology; TM5: 5% TM larvae, TM10: 10% TM larvae.

| Morphology | Basal diet | TM5 | TM10 | SEM | *P-value* |
| --- | --- | --- | --- | --- | --- |
| Duodenum Vh | 704.58 ^b^ | 790.07 ^a^ | 699.54 ^b^ | 10.06 | <0.001 |
| Duodenum Cd | 108.18 ^a^ | 83.34 ^b^ | 86.82 ^b^ | 2.77 | <0.001 |
| Jejunum Vh | 611.71 | 609.11 | 607.35 | 8.25 | 0.977 |
| Jejunum Cd | 82.45 ^b^ | 98.47 ^ab^ | 113.52 ^a^ | 4.17 | 0.008 |
| Ileum Vh | 493.06 ^b^ | 567.18 ^a^ | 494.47 ^b^ | 8.93 | <0.001 |
| Ileum Cd | 89.70 | 93.40 | 96.92 | 2.74 | 0.566 |

^a,b^ Values in the same row with no common superscript differ significantly (*P*<0.05)
